# A NAC family gene *PmNAC32* associated with photoperiod promotes flower induction in *Prunus mume*

**DOI:** 10.1093/hr/uhaf157

**Published:** 2025-06-23

**Authors:** Chengdong Ma, Pengyu Zhou, Yufan Ma, Wei Tan, Xiao Huang, Silas Segbo, Shahid Iqbal, Ting Shi, Zhaojun Ni, Zhihong Gao

**Affiliations:** Laboratory of Fruit Tree Biotechnology, College of Horticulture, Nanjing Agricultural University, No.1 Weigang, Xuanwu District, Nanjing 210095, China; Laboratory of Fruit Tree Biotechnology, College of Horticulture, Nanjing Agricultural University, No.1 Weigang, Xuanwu District, Nanjing 210095, China; Laboratory of Fruit Tree Biotechnology, College of Horticulture, Nanjing Agricultural University, No.1 Weigang, Xuanwu District, Nanjing 210095, China; Laboratory of Fruit Tree Biotechnology, College of Horticulture, Nanjing Agricultural University, No.1 Weigang, Xuanwu District, Nanjing 210095, China; Laboratory of Fruit Tree Biotechnology, College of Horticulture, Nanjing Agricultural University, No.1 Weigang, Xuanwu District, Nanjing 210095, China; Laboratory of Fruit Tree Biotechnology, College of Horticulture, Nanjing Agricultural University, No.1 Weigang, Xuanwu District, Nanjing 210095, China; Horticultural Science Department, North Florida Research and Education Center, University of Florida/IFAS, 155 Research Road, Quincy, FL 32351, USA; Laboratory of Fruit Tree Biotechnology, College of Horticulture, Nanjing Agricultural University, No.1 Weigang, Xuanwu District, Nanjing 210095, China; Laboratory of Fruit Tree Biotechnology, College of Horticulture, Nanjing Agricultural University, No.1 Weigang, Xuanwu District, Nanjing 210095, China; Laboratory of Fruit Tree Biotechnology, College of Horticulture, Nanjing Agricultural University, No.1 Weigang, Xuanwu District, Nanjing 210095, China

## Abstract

The photoperiod is essential to flower induction, and the exact timing of the process can be precisely regulated based on the relative duration of light and darkness. However, the mechanisms linking photoperiod and flower induction in woody plants remain largely unexplored. Using RNA-seq, we identified a photoperiod response factor *PmNAC32*, which is predominantly expressed in early-flowering varieties. Overexpression of *PmNAC32* in *Arabidopsis thaliana*, tobacco, and *Prunus mume* calli resulted in accelerated flowering. Binding and activation analyses revealed that *PmNAC32* can be directly suppressed by REVEILLE 1 (RVE1) and REVEILLE 3 (RVE3), implying that *PmNAC32* plays a role in the photoperiodic signaling pathway. Further studies established that PmNAC32 functions as a positive regulator of *CONSTANS-LIKE 5* (*COL5*) and a negative regulator of *CONSTANS-LIKE 4* (*COL4*). Interestingly, we identified two homologs of PmNAC32, namely PmNAC29 and PmNAC47. These three proteins can interact with each other and enhance the regulation of *PmCOL4* and *PmCOL5*. Although PmNAC29 and PmNAC47 can promote flower induction respectively, neither of them responded to the photoperiod. Thus, our results reveal a novel mechanism by which *PmNAC32* regulates flower induction in *Prunus mume*.

## Introduction

Following a phase of vegetative growth, flowering plants exhibit a continuous ability to perceive alterations in ambient elements and internal signals, thus ensuring their transition to the reproductive stage occurs under optimal circumstances [1]. Previous studies have comprehensively reviewed the molecular regulatory mechanisms of flowering in *Arabidopsis thaliana* [2, 3]. In contrast to annual plants, perennial trees have a distinct growth pattern characterized by prolonged vegetative growth spanning multiple years, followed by the initiation of intricate seasonal life processes, including flower induction, dormancy, dormancy release, and flowering [4–6]. As a deciduous tree in the Rosaceae family, *Prunus mume* trees must go through 4 years or a longer juvenile phase before they can flower. Specifically, flower induction mainly takes place in summer and autumn and bloom in the following early spring, which is affected by temperature and photoperiod [7–9].

The photoperiod pathway comprises three distinct components: photoreceptors, circadian clock proteins, and output pathways [10]. There is a well-defined mechanism in *Panicum hallii* about global gene expression, photoperiod interaction, phase shifts, and fine-scale contrasts of temporal dynamics under different photoperiod treatments [11]. External signals are sensed by the blue photoreceptor CRYPTOCHROME 1 (CRY1) and the red/far red photoreceptors PHYTOCHROME A (PhyA), PhyB, PhyD, and PhyE [12, 13]. *CONSTANS* (*CO*, also named *AtBBX1*), as the first B-box (BBX) gene, is a hub in the photoperiodic regulatory network [14]. The transcription of *CO* is inhibited by CYCLING DOF FACTOR 1–5 (CDF1–5), this inhibition is broken down by GIGANTEA (GI) and FLAVIN-BINDING, KELCH REPEAT, F-BOX 1 (FKF1) complex in the late afternoon [15–18]. At the same time, the stability of CO protein is regulated by E3 ubiquitin ligase CONSTITUTIVE PHOTOMORPHOGENIC 1 (COP1)-SUPPRESSOR OF PHYTOCHROME A 1 (SPA1) complex and photoreceptors [19–22]. As the length of daylight increases, the transcription level of CO is highly expressed and protein stability is strongest, which further stimulates the expression of *FT* and promotes flowering [23]. Other BBX family members also contribute to the regulation of photoperiod pathway. AtCOL3 (also named AtBBX4) can interact with AtBBX32 and targets *FT* in the presence of AtBBX32 to regulate the flowering time [24]. The direct interaction of CmBBX7 and CmBBX8 (orthologs of AtBBX7 and AtBBX8) improve the transcriptional level of *CmFTL1* and accelerate chrysanthemum flowering [25, 26]. Ectopic expression of peach *PpCO* (orthologs of *AtCO*) in *Arabidopsis co-2* mutants can rescue the late-flowering phenotype [27]. Besides, by analyzing the expression patterns at various flowering times, some *BBX* genes are strongly associated with flowering in *Platanus × acerifolia* [28]. Nevertheless, the mechanisms underlying woody plants remain unexplored.

*CIRCADIAN CLOCK-ASSOCIATED 1* (*CCA1*), *LATE-ELONGATED HYPOCOTYL* (*LHY*), and ortholog genes *REVEILLE*s (*RVE*s) are circadian clock genes that respond to the photoperiod [29, 30]. Moreover, RVE1 promotes the expression of *YUCCA8*, which participates in auxin biosynthesis [31]. RVE8 can target the promoter of the circadian clock gene *CCT MOTIF-CONTAINING RESPONSE REGULATOR PROTEIN* (*TOC1*) and activate its expression. However, Sorkin has demonstrated that this activation process can be inhibited at night by forming a ternary complex by NIGHT LIGHT-INDUCIBLE AND CLOCK-REGULATED 1 (LNK1) and either COLD-REGULATED 27 (COR27) or COR28 alongside RVE8 [32, 33]. During the late subjective day, *RVE8* and *PSEYDO-RESPONSE REGULATORS* (*PRR5*) establish a negative feedback loop [34]. All these mechanisms result to the early-flowering phenotype of *rve8* mutant. Recent reports have shown that RVEs have both light-dependent and red light-specific effects on the circadian system. The three members of the RVE family (*RVE4*, *RVE6*, and *RVE8*) share overlapping functions and are collectively regulated by CCA1 and LHY, contributing to circadian clock functions and flowering [32, 34–37].

The NAC family, as one of the largest transcription factor families, is universally characterized by two signature domains: a relatively conserved NAC domain and a highly variable transcriptional regulatory domain (TRD). The NAC domain, typically located at the N-terminal region of the protein, can be further categorized into five distinct subdomains (A–E) [38]. Studies have shown that NAC transcription factors are important in flowering regulation. Ning identified two NAC transcription inhibitors: *NAC050* and *NAC052*, which are linked to the histone demethylase JMJ14. This connection suggests that these NAC factors are involved in transcriptional inhibition and flowering time regulation [39]. The constitutive expression of the *Arabidopsis* NAC transcription factor LONG TROPHIC PERIOD 1 (LOV1) in switchgrass delays flowering time [40]. Additionally, JUNGBRUNNEN1 (JUB1) controls gibberellin (GA) and brassinosteroid metabolism, signal transduction, and decreased endogenous GA content in *Arabidopsis* transgenic plants. This reduction results in delayed flowering and male sterility [41]. *OsNAC2* affects the contents of endogenous abscisic acid and GA, leading to accelerated leaf aging and early flowering [42, 43]. OPEN STOMATA 1 (OST1)-VASCULAR PLANT ONE-ZINC FINGER 1 (VOZ1)-FT module plays a vital role in accelerating the flowering of tomatoes under drought stress [44]. Nevertheless, there is a scarcity of reports on the involvement of the NAC family in regulating plant flowering in response to photoperiodic signals.

In recent years, significant advancements have been made in studying *P. mume* [45–47]. At present, there is not enough research on the flower induction in *P. mume*. In this investigation, high-resolution transcriptome sequencing was utilized to examine the impact of photoperiod variation on the leaves in *P. mume*. A time-clustering identification method was employed to identify the genes associated with photoperiod. Our findings demonstrate that diminishing daylight hours trigger a progressive upregulation of *PmNAC32*, which establishes a feedback loop to counteract PmRVE1/3-mediated transcriptional suppression. Subsequently, PmNAC32 coordinates flower induction in *P. mume* by activating *PmCOL5* while simultaneously repressing *PmCOL4* transcription. Furthermore, PmNAC29 and PmNAC47 physically interact with PmNAC32 to synergistically enhance the promotion of flower induction. Based on these findings, we propose a novel regulatory mechanism where photoperiod-mediated control of flower induction in *P. mume* is achieved through the PmRVE1/3-PmNAC32-PmCOL4/5 transcriptional cascade. These findings open up new avenues for future studies on photoperiod.

## Results

### High-resolution time-spectral analysis of photoperiod response transcripts of *P. mume*

To initiate a comprehensive investigation of the photoperiod response genes in *P. mume*, we employed the RNA-seq technique to acquire precise temporal profiles of gene expression throughout the treatment of *P. mume* trees with short-day (SD) and long-day (LD) conditions. A total of 52 samples were taken for RNA sequencing from four *P. mume* trees, two LD trees and two SD trees, at 13 different time points from 0 to 48 h.

**Figure 1:**
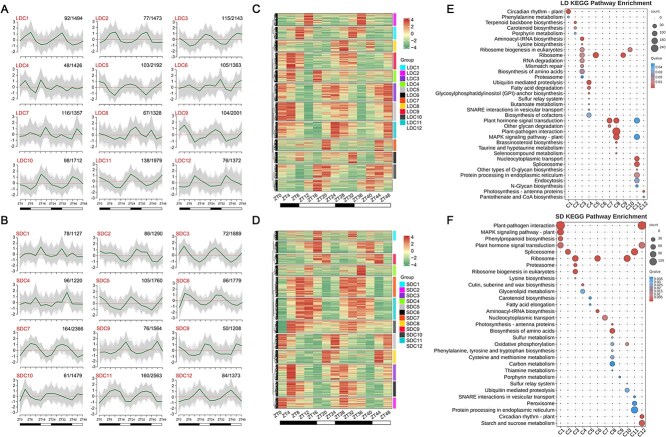
High-resolution temporal maps of *P. mume* leaf-related transcripts under LD and SD conditions. (A) and (B) Temporal clustering showed the gene expression changes under LD and SD conditions, which were divided into 12 clusters (LDC1–LDC12; SDC1–SDC12). The autoscaling log2(FPKM) values of transcripts in each cluster are shown. The number of transcription factors/genes. C, cluster. ZT means Zeitgeber time. White and black rectangles represent the photoperiod (light and dark phases). (C) and (D) Expression profiles of time-cluster genes under LD and SD conditions. (E) and (F) Functional clusters of KEGG functions enriched in differential clusters (LDC1–LDC12; SDC1–SDC12).

To provide further insight into the diversity of diurnal rhythm, we clustered the genes of LD and SD conditions into 24 clusters (represented as LDC1–LDC12 and SDC1–SDC12) based on hierarchical time series clustering (Fig. 1A and B). In contrast, the rhythmicity of gene expression in LDC4, LDC6, LDC8, SDC1, and SDC4 did not reach statistical significance. To comprehensively summarize the expression profiles, unsupervised hierarchical clustering was carried out across all clusters (Fig. 1C and D). The heatmap visualization clearly revealed that the majority of genes within each cluster exhibited expression patterns closely aligned with characteristic trend lines. Furthermore, phylogenetic tree branch analysis demonstrated evolutionary relatedness and pattern similarities among several gene clusters, suggesting potential functional associations between these molecular groups. Subsequently, Kyoto Encyclopedia of Genes and Genomes (KEGG) annotation was performed to allocate genes to functional categories within each cluster. Significantly enriched pathways were selected based on statistical criteria and visualized to represent the predominant biological processes associated with each cluster. Our analysis revealed that genes exhibiting diurnal rhythm characteristics were implicated in various pathways, including ubiquitin-mediated proteolysis, possibly due to its critical role in protein turnover and light-responsive regulatory mechanisms, and plant hormone signal transduction, for its central involvement in coordinating growth and stress adaptation under varying photoperiods (Fig. 1E and F). Notably, the distribution of the circadian rhythm pathway mostly occurs in two distinct clusters, namely LDC1 and SDC12. The confirmation of the LDC1 and SDC12 genes was also validated using Gene Ontology (GO) term enrichment analysis (Fig. S1A and B).

### Identification and expression patterns analysis of *PmNAC32*

**Figure 2:**
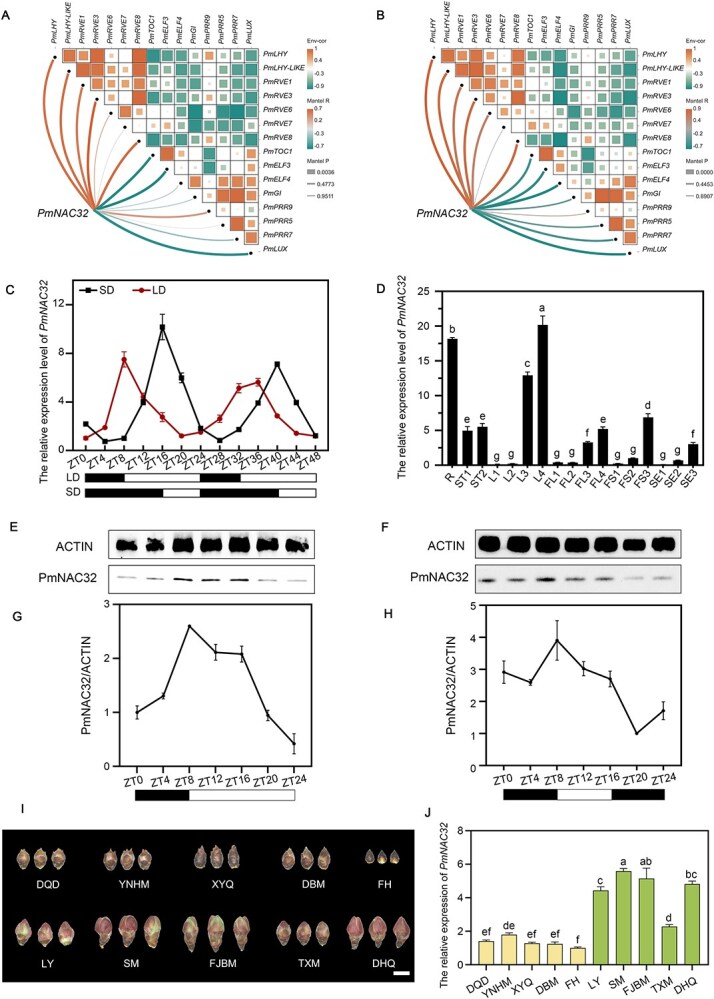
Identification and expression patterns of *PmNAC32*. (A) Correlation analysis between the *PmNAC32* and central oscillator genes under LD conditions. (B) Correlation analysis between the *PmNAC32* and central oscillator genes under SD conditions. (C) The transcriptional response to the varying photoperiod. The x-axis indicates the temporal point of sampling. Values are indicated as mean ± SE (*n* = 3). (D) qRT-PCR analysis of *P. mume* in different tissues at different periods. Error bars indicate the standard errors for three biological replicates. R, root. ST1, green stem. ST2, brown stem. L1, leaf bud. L2, tender leaf. L3, mature leaf. L4, yellow leaf. FL1, flower bud physiological differentiation stage. FL2, flower bud morphological differentiation stage. FL3, flower bud expansion stage. FL4, open flower. FS1, small size fruit. FS2, medium size fruit. FS3, big size fruit. FS4, mature fruit. SE1, pit of small fruit. SE2, pit of medium fruit. SE3, pit of big fruit. Values are indicated as mean ± SE (*n* = 3). (E–H) Western blot analysis of PmNAC32 protein in LD and SD conditions in *P. mume* leaves was performed using anti-PmNAC32 antibody. Image J was used for intensity analysis. Values are indicated as mean ± SE (*n* = 2). (I) Flower bud development of different varieties at the same time. The five late-flowering varieties above, the five early-flowering varieties below. DQD: ‘Da Qiandi’; YNHM: ‘Yunnan Hongmei’; XYQ: ‘Xi Yeqing’; DBM: ‘Da Baimei’; FH: ‘Fenghou’; LY: ‘Longyan’; SM: ‘Shamei’; FJBM: ‘Fujian Baimei’; TXM: ‘Tao Xingmei’; DHQ: ‘Da Heqing’. Bars in (I) = 4 cm. (J) The expression levels of *PmNAC32* were tested in 10 *P. mume* varieties. Statistical significance was determined using a one-way analysis of variance (ANOVA). Different letters indicate statistically significant differences (*P* < 0.05)

To better understand the diurnal rhythm chrono regulation in *P. mume*, we isolated the central oscillator genes already known from the transcriptome data (Fig. S2A). To understand the fundamental regulatory mechanisms of a thousand genes that follow the diurnal rhythm, we identified 45 transcription factors by analyzing the intersection between the LDC1 and SDC12 clusters (Table S3; Fig. S2B and C). Next, we analyzed the correlation between these transcription factors and central oscillator genes and found that one NAC family transcription factor LOC103325772 was significantly correlated with several central oscillator genes (Fig. 2A and B). This transcription factor was identified as PmNAC32 following a prior investigation conducted by Yao [48].

We used quantitative reverse transcription-polymerase chain reaction (qRT-PCR) to confirm the transcriptome findings to further elucidate the diurnal rhythm and higher SD expression level of *PmNAC32* (Fig. 2C). Next, qRT-PCR was employed to assess its expression profile in various developmental stages of *P. mume* tissues. The results indicated that *PmNAC32* exhibited higher expression level in mature tissues such as opened flowers, mature fruits, yellow leaves, and mature stems, and lower in young tissues (Fig. 2D). We then analyzed the promoter-β-glucuronidase (GUS) staining of *A. thaliana* plants transformed with the *PmNAC32*pro GUS reporter gene structure (*PmNAC32*pro::GUS). *PmNAC32*pro::GUS staining showed that *PmNAC32* expressed in every tissue except petals and stamens (Fig. S3A–E). Next, we measured the changes of PmNAC32 protein expression level in a day by western blot (Fig. 2E–H). As the dark period was extended, the protein bands became darker, while they lightened with the return of light. Meanwhile, after GUS staining the whole plant of *PmNAC32*pro::GUS lines, which were under the LD conditions, the color was darkest at ZT8 and lightest at ZT0/ZT24 (Fig. S3F).

To preliminarily elucidate the role of *PmNAC32* in the flower induction of *P. mume*, we analyzed its expression level in early-flowering varieties and late-flowering varieties (Fig. 2I). *PmNAC32* were more highly expressed in all five tested early-flowering varieties (Fig. 2J) and thus are probably associated with *P. mume* flower induction.

In summary, *PmNAC32* exhibited higher expression level in mature tissues and early-flowering varieties of *P. mume*, with its expression displaying distinct circadian rhythmicity.

### *PmNAC32* is a positive regulator of flower induction

**Figure 3:**
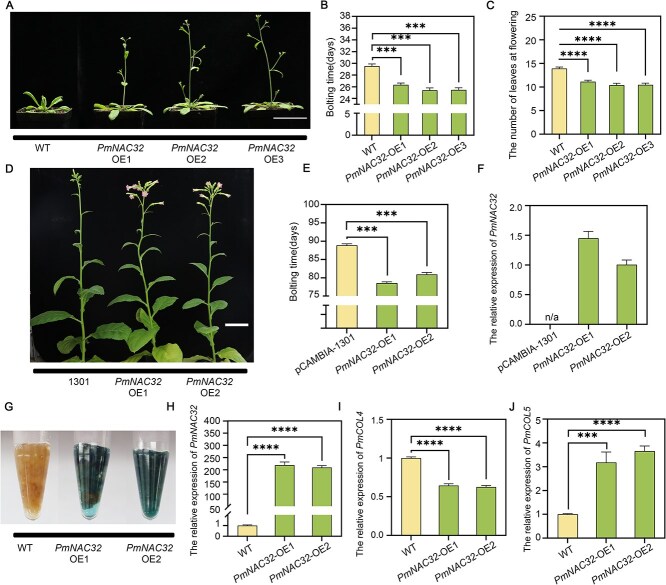
Overexpression of *PmNAC32* accelerates flowering time. (A) Flowering time phenotype of 26-day-old WT, *PmNAC32*-OE1-31, *PmNAC32*-OE2, and *PmNAC32*-OE3 under LD conditions. Bars in (A) = 5 cm. (B) Average bolting time of *A. thaliana* (OE lines and WT) shown in (A). Values are indicated as mean ± SE (*n* = 15). (C) Average number of leaves of *A. thaliana* (OE lines and WT). Values are indicated as mean ± SE (*n* = 15). (D) Flowering time phenotype of 95-day-old plants of the pCAMBIA1301-dual-35S empty vector control, *PmNAC32*-OE1 and *PmNAC32*-OE2 *N. tabacum* lines. Bars in (D) = 10 cm. (E) Average bolting time of *N. tabacum* (OE lines and pCAMBIA-1301 lines) shown in (D). Values are indicated as mean ± SE (*n* = 15). (F) The expression level of *PmNAC32* in *PmNAC32*-OE lines were assessed by qRT-PCR. Values are indicated as mean ± SE (*n* = 3). n/a means not applicable. (G) GUS staining of WT and *PmNAC32*-OE *P. mume* calli. (H), (I), and (J) The expression levels of *PmNAC32*, *PmCOL4*, and *PmCOL5* in *PmNAC32*-OE lines were assessed by qRT-PCR. Values are indicated as mean ± SE (*n* = 3). Stars above the bars show significant differences by Student’s test (^***^P < 0.001; ^****^P < 0.0001).

The establishment of a reliable genetic transformation method for *P. mume* remains a challenge, resulting in limited success in obtaining consistently transformed plants. To facilitate the investigation of the gene function of *PmNAC32*, we employed the pCAMBIA-1301 vector to induce overexpression of *PmNAC32* in *A. thaliana*. Subsequently, three distinct lines were selected to evaluate the impact of *PmNAC32* on the observable phenotypes of the plants (Fig. S4A–C). In the context of LD conditions, *PmNAC32* overexprssion (*PmNAC32*-OE) lines bolted significantly earlier than wild type (WT) when measured by both days and leaf number (Fig. 3A–C). As in LD conditions, *PmNAC32*-OE lines bolted significantly earlier than WT in SD conditions when measured by both leaf number and days (Fig. S4D–F). Subsequently, we proceeded to overexpress *PmNAC32* in *A. thaliana* using the estradiol-induced vector pER8. Following the application of the Estradiol (EST) treatment, we observed a notable elevation in *PmNAC32* expression levels and earlier flowering outcomes than the Dimethyl sulfoxide (DMSO) treatment (Fig. S5).

To gather additional evidence on early flowering, we overexpressed *PmNAC32* in *Nicotiana tabacum*. Subsequently, two lines were selected to observe the resulting phenotype (Fig. S4G and H). Under LD conditions, the overexpression of *PmNAC32* resulted in an accelerated flowering phenotype (Fig. 3D). Specifically, the *PmNAC32*-OE lines initiated the process of bolting 75 days after the seeds were sown, whereas the empty vector pCAMBIA-1301 lines began bolting at 90 days after seeding, indicating a difference of ~15 days (Fig. 3E and F).

To confirm the effect of *PmNAC32* on *P. mume*, we generated *PmNAC32* overexpression *P. mume* calli lines (Fig. 3G and H). Although the flowering phenotype could not be observed, we verified the expression trend of flowering-related genes, which are homologous genes of *Arabidopsis* flowering gene *AtCOL5* and *AtCOL4* by qRT-PCR (Fig. 3I and J), with *PmCOL5* upregulated and *PmCOL4* downregulated. Meanwhile, we obtained transient overexpression and silencing of *PmNAC32* in *P. mume* leaves using agroinfiltration. qRT-PCR results showed that the expression level of *PmNAC32* and *PmCOL5* was significantly increased after the overexpression of *PmNAC32* (Fig. S6A and B). In contrast, the expression level of *PmCOL4* was significantly reduced after the overexpression of *PmNAC32* (Fig. S6C). It is noteworthy that these genes exhibited an opposite expression trend in the *PmNAC32*-silenced lines (Figs. S6G–I). Taken together, these findings indicated that *PmNAC32* acts as a positive regulator of flowering.

### *PmNAC32* is the target gene of PmRVE1/3

**Figure 4:**
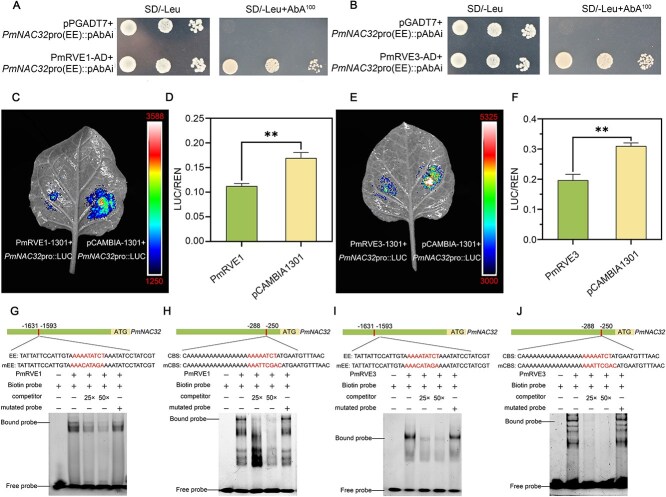
*PmNAC32* is the target gene of PmRVE1/3. (A) and (B) Interactions of PmRVE1 and PmRVE3 with the *PmNAC32*pro (EE) were detected in Y1H, respectively. AD represents the negative control. (C) and (D) The transcriptional regulation of the *PmNAC32*pro by PmRVE1 was investigated using the D-LUC assay. (E) and (F) The transcriptional regulation of the *PmNAC32*pro by PmRVE3 was investigated using the D-LUC assay. Luminescence signals were recorded by a CCD imaging system, and the pseudo-color bar illustrates the intensity scale. Empty vector pCAMBIA-1301 is the negative control, and values are indicated as mean ± SE (*n* = 3). Student’s test identified significant differences from the control (^**^P < 0.01). (G) and (H) His-PmRVE1 and His-PmRVE3 fusion proteins can bind EE-box in the *PmNAC32*pro. (I) and (J) His-PmRVE1 and His-PmRVE3 fusion proteins can bind CBS-box in the *PmNAC32*pro. 5’ FAM-labeled DNA probes were used. Same but unlabeled DNA probes were used as competitors, different but unlabeled DNA probes were used as mutators.

Based on our analysis, it can be inferred that the transcriptional level of *PmNAC32* primarily accumulates during nighttime, as indicated by the diurnal rhythm expression pattern. Additionally, numerous photoresponsive elements were identified in the promoter sequence of *PmNAC32*, such as G-box [49], circadian [50], EE-box [51, 5,2], and CBS-box [53] (Fig. S7). Also, an earlier report noted that LHY/CCA1 primarily functions as a suppressor by binding to the CCA1-binding site (CBS) and Evening Element (EE) within the promoter sequence [54]. To conduct a thorough screening of potential upstream *LHY/CCA1* homolog genes, we employed the yeast one-hybrid (Y1H) assay to screen PmLHY, PmLHY-like, and PmRVEs (PmRVE1, PmRVE3, PmRVE6, PmRVE7, and PmRVE8). In light of the unimpeded autonomous activation exhibited by *PmNAC32*pro (CBS), the experiment was conducted to identify the genes capable of binding to *PmNAC32*pro (EE) (Fig. S8A). The findings indicate that PmRVE1 and PmRVE3 can directly bind to *PmNAC32* (Fig. 4A and B; Fig. S8B). Subsequently, the experimental conclusion was further substantiated by employing dual-luciferase (D-LUC) and electrophoretic mobility shift assay (EMSA) techniques (Fig. 4C–J). Taken together, our findings collectively indicate that the proteins PmRVE1 and PmRVE3 can directly suppress the expression of *PmNAC32*.

Additionally, due to the presence of feedback regulation in many genes in the photoperiod pathway [52, 55], we performed qRT-PCR to detect the expression level of *PmLHY*, *PmRVE1*, and *PmRVE3* from the calli and leaves of *PmNAC32*-OE and pTRV-*PmNAC32* (Fig. S6D–F, J–L; Fig. S9). The results showed that *PmLHY* did not change significantly in *PmNAC32*-OE and pTRV-PmNAC32 lines, but the expression level of *PmRVE1* and *PmRVE3* decreased significantly in *PmNAC32*-OE lines and increased in pTRV-*PmNAC32* line.

### PmNAC32 promotes flower formation by directly binding the promoter of *PmCOL4* and *PmCOL5*

**Figure 5:**
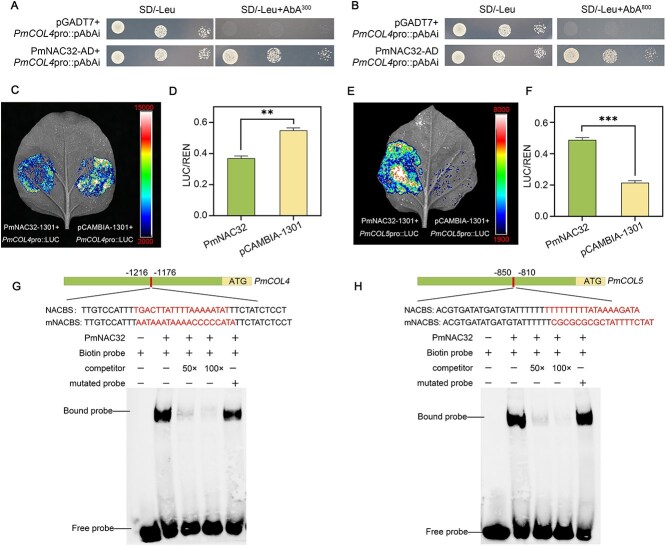
PmNAC32 directly bind the promoters of *PmCOL4* and *PmCOL5*. (A) and (B) Interactions of PmNAC32 with the *PmCOL4* and *PmCOL5* promoter were detected in Y1H, respectively. AD represents the negative control. (C) and (D) D-LUC experiments were performed with PmNAC32 protein and *PmCOL4* promoter. (E) and (F) D-LUC experiments were performed with PmNAC32 protein and *PmCOL5* promoter. The empty vector pCAMBIA-1301 is the negative control, and values are indicated as mean ± SE (*n* = 3). Student’s test identified significant differences from the control (**P < 0.01; ***P < 0.001). (G) and (H) His-PmNAC32 fusion protein can bind 4E1 and 5E1 in the *PmCOL4* and *PmCOL5* promoter. 5’ Biotin-labeled DNA probes were used. Same but unlabeled DNA probes were used as competitors. Different but unlabeled DNA probes were used as mutators. Luminescence signals were recorded by a CCD imaging system, and the pseudo-color bar illustrates the intensity scale.

Given the responsiveness of *PmNAC32* to variations in photoperiod and its ability to expedite the process of flower induction, our hypothesis posits that BBX family genes may serve as downstream genes of PmNAC32. The genes *AtCOL4* and *AtCOL5* were previously regarded as suppressor and promoter genes, respectively, in relation to flowering time [56, 57]. Promoter analysis detected NACBS in the promoter of *PmCOL4* and *PmCOL5*. PmNAC32 was inserted into the AD vector, and subsequent Y1H experiments demonstrated the direct binding capability (Fig. 5A and B). *In vivo* experiments with co-expression of PmNAC32 and *PmCOL4*pro::LUC in *Nicotiana benthamiana* showed that PmNAC32 inhibited the expression of *PmCOL4* (Fig. 5C and D). While co-expression of PmNAC32 and *PmCOL5*pro::LUC in *N. benthamiana* showed that PmNAC32 activated the expression of *PmCOL5* at the transcriptional level (Fig. 5E and F). *In vitro* EMSA revealed that the entire PmNAC32 protein binds to two sites on the *PmCOL5* promoter (5E1, 5E2) and four sites on *PmCOL4* promoter (4E1, 4E2, 4E3, 4E4) (Fig. 5G and H; Fig. S10).

Together, these results suggest that PmNAC32 directly influences their transcription of *PmCOL4* and *PmCOL5*.

### Overexpression of *PmCOL4* and *PmCOL5* in *A. thaliana*

**Figure 6:**
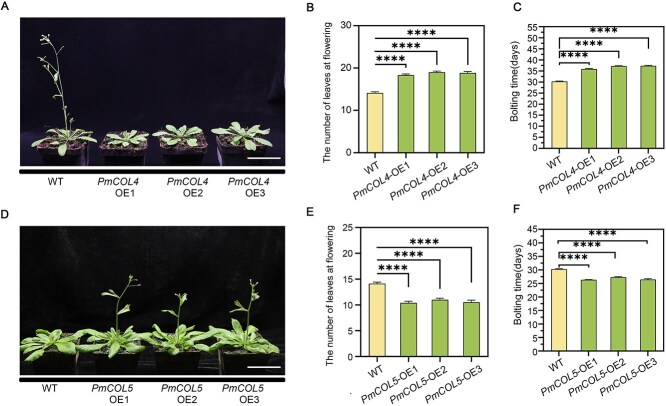
Overexpression of *PmCOL4* and *PmCOL5*, influences flowering time in *A. thaliana* under LD conditions. (A) Flowering time phenotype of 35-day-old WT, *PmCOL4*-OE1, *PmCOL4*-OE2, and *PmCOL4*-OE3. Bars in (A) = 5 cm. (B) Average bolting time of *A. thaliana* (OE lines and WT) shown in (A). Values are indicated as mean ± SE (*n* = 15). (C) Average number of leaves of *A. thaliana* (OE lines and WT). Values are indicated as mean ± SE (*n* = 15). (D) Flowering time phenotypes of 27-day-old plants of the WT, *PmCOL5*-OE1, *PmCOL5*-OE2, and *PmCOL5*-OE3. Bars in (D) = 5 cm. (E) Average bolting time of *A. thaliana* (OE lines and WT) shown in (D). Values are indicated as mean ± SE (*n* = 15). (F) Average number of leaves of *A. thaliana* (OE lines and WT). Values are indicated as mean ± SE (*n* = 15). Stars above the bars show significant differences by Student’s test (^***^P < 0.001; ^****^P < 0.0001).

Although *COL4* and *COL5* homologous genes have been shown to be involved in flowering regulation in other species, they have not been validated in *P. mume*. To facilitate the investigation of the gene function of *PmCOL4* and *PmCOL5*, transgenic plants were obtained. Subsequently, three distinct lines were selected to evaluate the impact of *PmCOL4* and *PmCOL5*, respectively, on the observable phenotypes of the plants (Fig. S11). In the context of LD conditions, *PmCOL4*-OE lines bolted significantly later than WT when measured by both days and leaf number (Fig. 6A–C). While *PmCOL5*-OE lines bolted significantly earlier than WT in short days when measured by both leaf number and days (Fig. 6D–F).

### The overexpression of *PmNAC32* homologous genes *PmNAC47* and *PmNAC29* accelerated the flowering of *A. thaliana*

**Figure 7:**
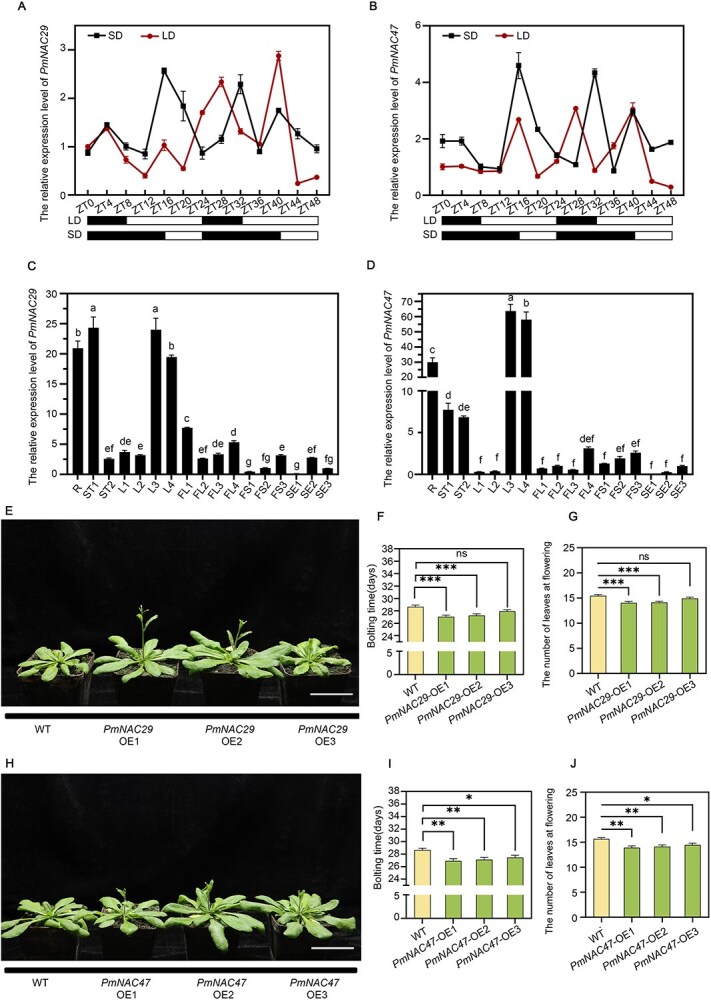
Analysis of expression patterns and LD phenotype of *PmNAC29* and *PmNAC47*. (A) and (B) The transcriptional pattern of *PmNAC29* and *PmNAC47* does not respond to the varying photoperiod. The x-axis indicates the temporal point of sampling, and white and black rectangles represent the photoperiod (light and dark phases). Values are indicated as mean ± SE (*n* = 3). (C) and (D) qRT-PCR analysis of *PmNAC29* and *PmNAC47* in different tissues at different periods. Values are indicated as mean ± SE (*n* = 3). (E) Flowering time phenotype of 28-day-old WT and *PmNAC29*-OE lines. Bars in (E) = 5 cm. (F) Average bolting time of wild-type plants and *PmNAC29*-OE lines. Values are indicated as mean ± SE (*n* = 15). (G) Average number of leaves of *A. thaliana* (OE lines and WT). Values are indicated as mean ± SE (*n* = 15). (H) Flowering time phenotype of 29-day-old WT and *PmNAC47*-OE lines. Bars in (H) = 5 cm. (I) Average bolting time of wild-type plants and *PmNAC47*-OE lines. Values are indicated as mean ± SE (*n* = 15). (J) Average number of leaves of *A. thaliana* (OE lines and WT). Values are indicated as mean ± SE (*n* = 15). Stars above the bars show significant differences by Student’s test (*ns* > 0.05; ^*^P < 0.05; ^**^P < 0.01; ^***^P < 0.001; ^****^P < 0.0001). Statistical significance was determined using ANOVA. Different letters indicate statistically significant differences (*P* < 0.05)

By employing amino acid sequence alignment, we have identified two genes, namely *PmNAC29* and *PmNAC47*, in *P. mume* that exhibit a high degree of similarity with *PmNAC32* in their conserved regions (Fig. S12). However, *PmNAC29* and *PmNAC47* exhibited nonrhythmic expression profiles under both LD and SD conditions (Fig. 7A and B). The tissue expression patterns of the two were further investigated using qRT-PCR. The findings indicated that the three genes exhibited high expression level in flowers and yellow leaves. However, in contrast to *PmNAC32*, *PmNAC29* and *PmNAC47* displayed the highest expression in stems (Fig. 7C and D).

To enhance the examination of the functional association between *PmNAC29* and *PmNAC47* with *PmNAC32*, we acquired *PmNAC29*-OE lines and *PmNAC47*-OE lines in *A. thaliana* (Fig. S13). The transgenic lines *PmNAC29*-OE and *PmNAC47*-OE exhibited a precocious flowering phenotype when subjected to both LD and SD conditions (Fig. 7E–J; Fig. S13). To summarize, the proteins PmNAC29, PmNAC47, and PmNAC32 exhibit comparable roles in promoting the process of flowering. Notably, only the expression of *PmNAC32* aligns with the diurnal rhythm.

### PmNAC32 can physically interact with PmNAC29 and PmNAC47 and the complex synergistically regulate *PmCOL4* and *PmCOL5* expression

Given that previous studies have demonstrated NAC family transcription factors coordinate plant growth and development through mutual protein interactions [58], it is reasonable to hypothesize the existence of physical interactions among these three proteins. In order to evaluate the validity of this theory, we investigated the transcriptional activation potential of these three proteins. When assayed in synthetic dropout medium lacking leucine, tryptophan, histidine, and adenine, all three fusion proteins demonstrated strong transactivation activity as evidenced by robust yeast growth. Truncation analysis revealed that all of the transcriptional activation domains reside within C-terminal regions: residues 192-320aa in PmNAC32, 179-302aa in PmNAC29, and 184-315aa in PmNAC47 (Fig. S14).

**Figure 8:**
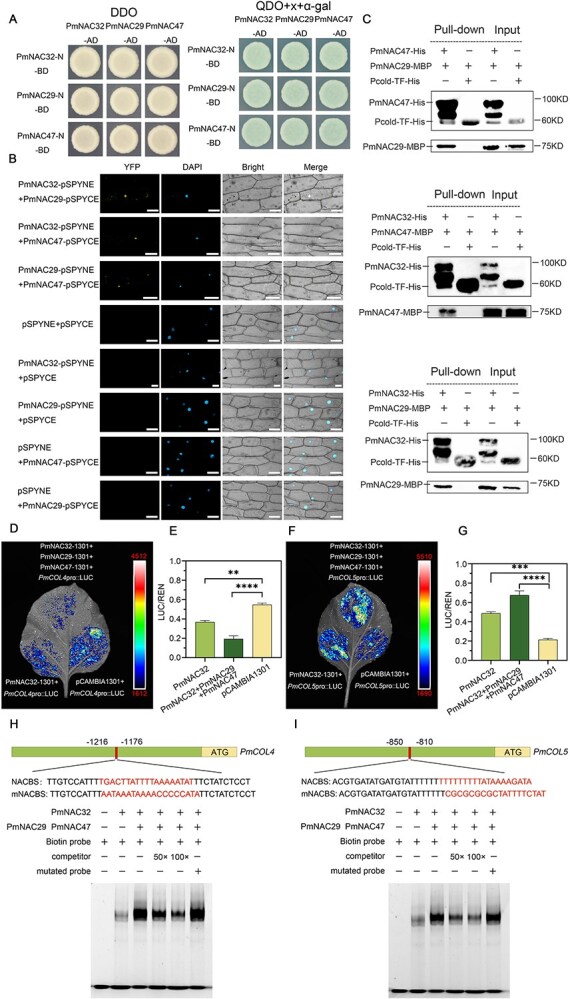
PmNAC32, PmNAC29, and PmNAC47 interact with each other. (A) Analysis of interactions between PmNAC32, PmNAC29, and PmNAC47 by Y2H. (B) BiFC assay. YFP: images obtained through the YFP fluorescence channel; DAPI: images obtained through the DAPI channel; Bright: images obtained through bright light; Merged: composite overlay images; bars = 100 μm. (C) Pull-down assays. His empty vector, MBP-PmNAC29, and MBP-PmNAC47 were used as controls. The arrows showed the target protein locations. (D) and (E) D-LUC experiments were performed with the complex and *PmCOL4* promoter. (F) and (G) D-LUC experiments were performed with the complex and *PmCOL5* promoter. The empty vector pCAMBIA-1301 is the negative control, and values are indicated as mean ± SE (*n* = 3). Student’s test identified significant differences from the control (^**^P < 0.01; ^***^P < 0.001, ^****^P < 0.0001). (H) and (I) The complex can bind 4E1 and 5E1 in *PmCOL4* and *PmCOL5* promoters more strongly. 5’ Biotin-labeled DNA probes were used. Same but unlabeled DNA probes were used as competitors. Different but unlabeled DNA probes were used as mutators.

Next, we selected PmNAC32-N, PmNAC47-N, and PmNAC29-N, which lacked the self-activating domain, as bait and fused PmNAC32, PmNAC47, and PmNAC29 with AD as prey for verification. The experimental findings demonstrated that the three proteins can self-assemble into homologous complexes and build heteromeric protein complexes, including two of the proteins (Fig. 8A).

Additionally, bimolecular fluorescence complementation assay (BiFC) was employed to validate the findings. The experimental setup involved the co-transformation of onion epidermal cells with three different combinations of plasmids: PmNAC32-nYFP and PmNAC29-cYFP, PmNAC32-nYFP and PmNAC47-cYFP, and PmNAC29-nYFP and PmNAC47-cYFP (Fig. 8B). When utilizing confocal microscopy, a pronounced fluorescent signal is produced within the nucleus. The negative control group did not exhibit any fluorescent signal. The His pull-down further illustrates the interaction between these three subunits (Fig. 8C). The above findings demonstrate that PmNAC32, PmNAC29, and PmNAC47 can engage in physical interactions *in vivo* and *in vitro*.

To further investigate the mechanism underlying the interactions of PmNAC32, PmNAC29 and PmNAC47, the D-LUC assay was used to measure their influence on *PmCOL4* and *PmCOL5* transcription. Compared with the empty vector pCAMBIA-1301, both PmNAC32 and interactions could inhibit LUC activity under the *PmCOL4* promoter, and the complex demonstrated greater inhibition potential than that of PmNAC32 alone (Fig. 8D and E). Similarity, compared with the empty vector pCAMBIA-1301, both PmNAC32 and the complex could activate LUC activity under the *PmCOL5* promoter, and the complex demonstrated greater activation potential than that of PmNAC32 alone (Fig. 8F and G). The EMSA assay was used to further demonstrate the function of the complex. From the result we can observe that the complex has deeper binding bands than the PmNAC32 protein alone (Fig. 8H and I).

### PmNAC32 homologs do not have circadian expression patterns in *A. thaliana*

**Figure 9:**
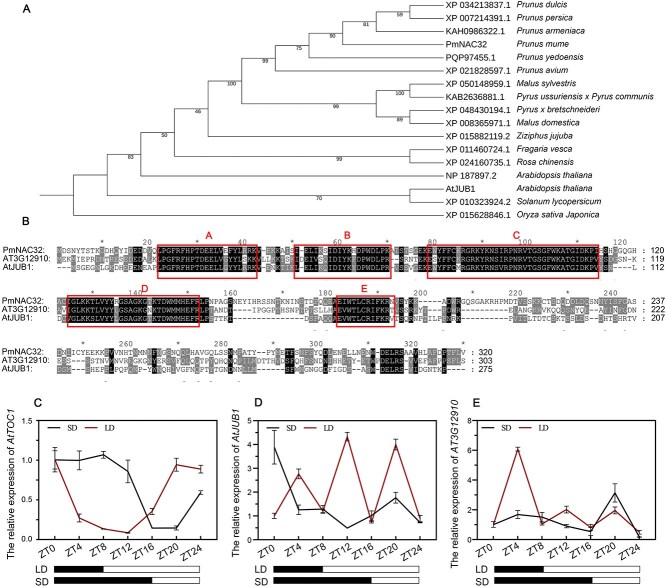
PmNAC32 homologous proteins AT3G12910 and AtJUB1 and their rhythmic expression patterns (A) Phylogenetic and evolutionary analysis of PmNAC32 homologous proteins in rice, *Arabidopsis* and Rosaceae family. Iqtree software is used to build Maximum-likelihood tree based on JTT + F + I + G4 model with a boostrap of 1000. The homologous sequence XP 015628846.1 of monocotyledonous rice was taken as outgroup, and all sequences were derived from the NCBI database. The percentage of trees where related taxa are clustered together is shown next to the branches. (B) The amino acid sequences of PmNAC32, AT3G12910, and AtJUB1 were aligned by Clustal X program. The amino acid residues conserved among the proteins are highlighted in black. The different amino acid residues are indicated with an asterisk*. The frame indicated the conserved NAM domain. (C) *AtTOC1* (D) *AtJUB1* (E) AT3G12910. The impact of photoperiod on transcriptional activity was evaluated by qRT-PCR. Values are indicated as mean ± SE (*n* = 3).

An evolutionary tree analysis was conducted on PmNAC32 protein in *A. thaliana*, rice, and various Rosaceae species to comprehend the background of *PmNAC32* study (Fig. 9A). In *A. thaliana*, two genes have high protein similarity with PmNAC32, namely AtJUB1 and AT3G12910 (NP 187897.2) (Fig. 9B). In addition, we employed qRT-PCR to acquire precise temporal profiles of *AtJUB1* and AT3G12910 expression throughout the treatment of *A. thaliana* lines with SD and LD conditions. Samples were taken from six *A. thaliana* plants, three LD and three SD lines, at 7 different time points from 0 to 24 h. *AtTOC1* was selected as a positive control. The results showed that *AtJUB1* and AT3G12910 do not respond to photoperiod (Fig. 9C–E).

## Discussion

Plants possess the ability to utilize their circadian clock to anticipate forthcoming alterations in environmental factors, such as light and temperature. This enables them to accurately synchronize the time of their flowering transition with favorable environmental conditions. Previous research has indicated that the regulation of seasonal growth in woody perennials is influenced by photoperiodic signals detected in leaves. This detection process leads to the cessation of tree growth when daylight gradually decreases and falls below a threshold that allows growth. As a result, apical elongation is halted and the differentiation of flower buds is promoted [59], but the molecular mechanism is still elusive.

As one of the earliest flowering woody perennial plants in spring, *P. mume* is a deciduous fruit tree and exhibits unique phenological characteristics: its flower induction occurs from June to September, followed by a dormancy period that terminates with winter-chilling requirements. This distinctive flowering rhythm, featuring seasonal bud formation and exceptionally early bloom, establishes *P. mume* as an excellent model system for studying flowering. Moreover, the availability of the high-quality reference genomes [45,46,60] and extensive germplasm resources containing diverse varieties provides exceptional opportunities for molecular investigations into flowering mechanisms and variety-specific trait analyses. However, there is uncertainty regarding how photoperiod regulates flower induction. Consequently, we identified several genes with diurnal rhythm in *P. mume* (Fig. 1). The expression patterns of central oscillator genes are the same as those in *A. thaliana* (Fig. S2A). This discovery provided evidence that aligns with Zdepski’s argument that clock system genes exhibit conservation across various plant species [61].

In recent years, flowering integrators in *P. mume* (*PmFT*, *PmSOC1*, *PmLFY*, and *PmTFL*) have gradually been identified [62–65]. Recent studies have demonstrated that PmRGL2 and PmFRL3 synergistically regulate flowering in *P. mume* through coordinated control of *PmSVP* and *PmSVP-like* genes [66]. In this study, we discovered 45 transcription factors compatible with diurnal rhythm expression patterns from RNA-seq data (Fig. S2B and C). Among the 45 transcription factors, *PmNAC32* emerged as a prime candidate due to its co-expression with central oscillator genes (Fig. 2A and B). This tight coupling suggests *PmNAC32* may act as an output module translating circadian rhythms into developmental signals. Further studies found that *PmNAC32* exhibits photoperiod-responsive expression, as evidenced by its diurnal rhythm at both transcriptional and protein levels (Fig. 2C, E–H), and is significantly upregulated in early-flowering varieties compared to late-flowering varieties (Fig. 2I and J).

The overexpression of *PmNAC32* in *A. thaliana*, *N. tabacum*, and *P. mume* calli and leaves promoted flowering and altered the transcriptional profiles of flowering regulatory genes (Fig. 3; Figs S5, S6, and  S9). Conversely, *PmNAC32*-silenced *P. mume* leaves exhibited reversed expression changes in these flowering regulatory genes (Fig. S6). This finding illustrates the capacity of *PmNAC32* to promote plant flower induction. 

Previous studies have found that the NAC family can participate in plant development (such as seed development, root development, flowering, leaf senescence, fruit ripening, and stress response) [38]. Among them, the mechanism of NAC family regulation of flowering is mainly focused on hormone signals such as gibberellin and ethylene [67, 68]. There are few reports on the involvement of the NAC family in the diurnal rhythm pathway. Zhu found that *ANAC017* can regulate the mitochondrial retrograde response, which interacts with the circadian clock regulator in *Arabidopsis*, further research on its regulatory mechanism is needed [69]. In our research, the identification of EE-box and CBS-box in the *PmNAC32* promoter, coupled with their direct suppressing by PmRVE1/3, established a molecular link between circadian clock and PmNAC32 activation (Fig. 4). Based on these findings, we propose a photoperiod-regulated molecular mechanism governing flower induction in P. mume. During March to June when daylight duration progressively increases, the expression of *PmNAC32* is suppressed by both extended photoperiod and PmRVE1/3 transcriptional repressors, coinciding with leaf bud germination and active vegetative growth. Conversely, from June to September as daylight hours decrease, *PmNAC32* expression gradually elevates and constructs a feedback regulatory mechanism that counteracts PmRVE1/3-mediated suppression. This photoperiod-responsive transcriptional dynamics ultimately promotes the initiation of flower induction in autumn.

*Arabidopsis thaliana*’s ability to blossom has been demonstrated to be influenced by *AtCOL4* and *AtCOL5* [56, 57]. We hypothesized that *PmNAC32* may be targeting *PmCOL4* and *PmCOL5*, which is one way in which *PmNAC32* alters the timing of plant flowering. Previously, mutation analysis combined with the EMSA method was used to define the NAC family binding sequence, and the results showed that WNNYBTNNNNNNAMGNHW and TTRCGT were possible NACBSs [70, 71]. Through Y1H and EMSA experiments, *PmNAC32* can directly bind NACBS on the promoters of *PmCOL5* and *PmCOL4* (Fig. 5A, B, G, H). It was found through LUC experiments that *PmNAC32* could promote the expression of *PmCOL5* but inhibited the expression of *PmCOL4*, indicating that *PmNAC32* could affect the flower induction of *P. mume* through both *PmCOL5* and *PmCOL4* (Fig. 5C–F). Simultaneously, we did not find EE-box and CBS-box in the promoter region of *PmCOL4* and *PmCOL5*, indicating that these two genes are not direct target candidate genes for LHY/CCA1 and REVEILLEs. In order to make it more convincing that PmNAC32 regulates *PmCOL4* and *PmCOL5* to accelerate plant flowering, we conducted heterologous expression of *PmCOL4* and *PmCOL5* in *A. thaliana*. The results showed that *PmCOL4* significantly delayed the flowering time, while *PmCOL5* significantly advanced the flowering time (Fig. 6A–F). Therefore, we speculate that *PmNAC32* may be a key gene that regulates flower induction in response to photoperiodic signals.

NAC family genes often coordinately regulate downstream genes [72, 73]. Homologous proteins of PmNAC32, PmNAC29, and PmNAC47 exhibit similar flowering-promoting abilities and interact with PmNAC32 (Fig. 7E–J; Fig. 8A–C), but do not respond to the photoperiod at the transcriptional level as well as deletion of EE-box on their promoters (Fig. 7A and B). These results suggest that *PmNAC29* and *PmNAC47* may assist *PmNAC32* promoting flowering. So, we transfected PmNAC32, PmNAC29, and PmNAC47 into *N. benthamiana* leaves together, and the results showed that the complex of NACs has stronger influence in downstream genes than PmNAC32 alone (Fig. 8D–G). Consistent with the results of D-LUC experiments, EMSA also demonstrated that the binding of the complex to downstream genes was stronger than that of PmNAC32 single protein (Fig. 8H and I). By exchanging the promoter and CDS region of homologous genes *ANAC060*, *ANAC040*, and *ANAC089*, Song identified that the phenotypic differences among the native genes are potentially driven by their distinct transcriptional patterns [74]. Whereas the proteins exhibit comparable functional traits, the regulation of the genes implicated diverges significantly due to alterations in the promoter regions. Therefore, we implied that *PmNAC32* acts as the critical gene in photoperiodic regulation of flower induction, while *PmNAC29* and *PmNAC47* act as its auxiliary genes due to differences in promoter *cis*-elements.

Unlike annual herbaceous species such as *Arabidopsis*, *P. mume* exhibits perennial woody growth with annual flowering recurrence. Investigating the flower induction in *P. mume* will provide insights into the rate, quantity, and quality of flower induction in perennial woody fruits. In *Arabidopsis*, *JUB1*, the homologous gene of *PmNAC32*, integrates interactions between the gibberellin and brassinosterioid hormones, resulting in late flowering and leaf senescence [41, 75–77]. The observed differences in circadian expression patterns between *PmNAC32* and *AtJUB1*/AT3G12910 may reflect evolutionary adaptations to distinct life-history strategies in woody and herbaceous plants (Fig. 9). Woody species such as *P. mume*, characterized by extended lifespans and intricate seasonal flowering requirements, likely evolved robust circadian control of genes like *PmNAC32* to synchronize critical flower induction with environmental cues photoperiod. In contrast, herbaceous plants like *A. thaliana*, optimized for rapid life cycles and immediate stress responses, appear to prioritize direct environmental or developmental triggers. In Chinese flowering cabbage, *BrJUB1* act as a positive regulator in leaf senescence, which is different with *AtJUB1* [78]. Phenotypic differences suggest that *NAC32* may regulate plant flowering through different pathways in different species. These distinctive biological traits and genetic mechanisms position *P. mume* as a pivotal model for bridging the knowledge gap between herbaceous and woody perennial plants, offering novel frameworks to decipher perennial developmental plasticity and adaptive evolution in plant systems.

## Conclusion

**Figure 10:**
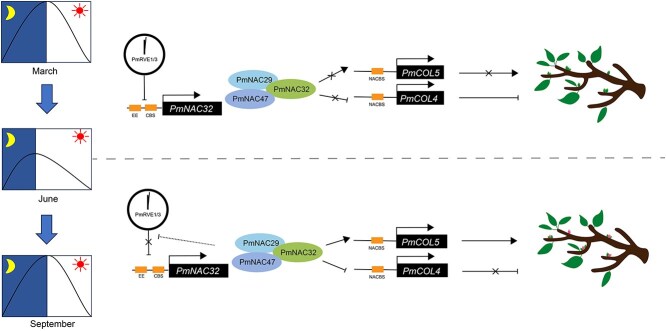
The model diagram illustrates that *PmNAC32* responds to photoperiod signals and promotes floral bud differentiation in *P. mume* through the PmRVE1/3-PmNAC32-PmCOL4/5 transcriptional cascade from June to September, as daylight duration gradually shortens.

In conclusion, our study identified the NAC family transcription factor PmNAC32, which expression level showed diurnal rhythm and worked as a bridge for the photoperiod to forecast photocycle signals promoting flower induction through the PmRVE1/3-PmNAC32-PmCOL4/5 pathway (Fig. 10). Meanwhile, PmNAC32 can also form a protein complex with PmNAC29 and PmNAC47 to promoter flower induction. These findings provide novel insight into the molecular mechanism of *PmNAC32* on flower induction regulation with the photoperiod system in perennial woody plants.

## Materials and methods

### Plant materials and growth conditions

Three-year-old ‘Longyan’ *P. mume* tree seedlings were cultivated in a substrate composed of perlite, vermiculite, and soil at 24°C in a thermostatic glasshouse in Nanjing Agricultural University. At 13 different time points, LD (16 h light:8 h dark) and SD (8 h light:16 h dark) were used to collect leaves at 4-h intervals for 48 h. Each time, total RNA was extracted from about three leaves from three branches of each regenerated tree and immediately frozen with liquid nitrogen. Samples of multiple *P. mume* varieties and the tissue of *P. mume* variety ‘Longyan’ were procured from the National Field Genebank for *P. mume* for later usage. The stage of full bloom period of early-flowering varieties (LY, SM, FJBM, TXM, DHQ) is in late January and early February, while that of late-flowering varieties (DQD, YNHM, XYQ, DBM, FH) is in late February and early March. Harvested tissues were promptly immersed in liquid nitrogen for rapid freezing and maintained at −80°C. *Arabidopsis thaliana* lines were grown under LD conditions for 12 days. Then they were transferred to growth chambers set to a 16-h or 8-h light photoperiod and maintained at 24°C. *Nicotiana tabacum* and *P. mume* calli were grown under LD conditions.

### Identification photoperiod response genes of *P. mume* by RNA-seq

In the RNA-seq study employing two complete 24-h cycles (48-h sampling), each time point across both cycles contains duplicate biological replicates, yielding four independent measurements per temporal phase (2 biological replicates × 2 cycles). This multicycle replication strategy compensates for conventional biological replication by providing both technical reproducibility between cycles and biological variation assessment within cycles. To accomplish RNA-seq, the Illumina HiSeq 4000 platform was utilized (BGI Genomics, Shenzhen, China). Raw reads (fastq format) were pruned and filtered by internal SOAPnuke software (BGI Genomics, Shenzhen, China). After processing, 2.214 billion high-quality reads (average Qphred >20 = 97.68%) and 332.06 Gb of clean bases were generated. The clean reads were aligned to the *P. mume* genome (GCF_000346735.1_*P. mume*_V1.0) with the HISAT software [79, 80]. Localization genes were used for identification and cluster analysis. The cluster analysis was based on the Mfuzz method. The replicates showed high concordance (correlation coefficient > 0.9). Genes that follow photoperiodic regulation were screened under LD and SD conditions.

### Isolation and sequence analysis of *PmNAC32*

Gene-specific primers were designed in reference to the sequence LOC103325772 of *P. mume* and amplified (Table S1); the product was separated on agarose gel electrophoresis. Following their recovery, the selected fragments were ligated using the pClone007 Blunt vector (Tsingke Biotechnology, Beijing, China). The potential *cis*-elements in the promoter region (2000 bp upstream) of the *PmNAC32* gene were identified using the PlantCARE database.

### GUS histochemical staining

GUS staining experiments were performed according to the established protocol with minor modifications [81]. Fresh plant tissues were collected and rinsed with distilled water. Samples were vacuum-infiltrated in GUS staining solution (0.1 M sodium phosphate buffer, pH 7.0, containing 1 mM X-Gluc, 0.5 mM potassium ferrocyanide, and 0.5 mM potassium ferricyanide) followed by 24 h of incubation at 37°C in darkness. Chlorophyll and nonspecific staining were eliminated through sequential ethanol dehydration (70%, 90%, and 100%) until complete tissue decolorization.

### Overexpression vector construction and genetic transformation

The homologous recombinant primer (Table S1) was designed based on the overexpression vector pCAMBIA1301-dual-35S (pCAMBIA-1301) and pER8 sequences, and the open reading frame (ORF) region was constructed into the vector and introduced into *Agrobacterium tumefaciens* strain GV3101. Single bacteria were isolated into 50 ml of Luria–Bertani broth containing 50 mg/l Kanamycin and Rifampicin and cultured at 220 rpm in an incubator at 28°C until optical density at 600 nm of 0.6 was achieved. After centrifugation, the cell pellet was resuspended in 50 ml of hormone-free, uncontrolled pH Murashige and Skoog (MS) liquid medium for 30 min; *N. tabacum* transformation was then performed by the leaf disk method. After the resistant seedlings were isolated, qRT-PCR was performed to identify positive transgenic lines. The genetic transformation of *A. thaliana* was performed by the flower soaking method, and transgenic positive lines were screened with one-half MS medium containing 30 mg/l hygromycin. The expression level of *PmNAC32* in transgenic positive lines was detected by qRT-PCR.

The *P. mume* calli infection method was the same as the *N. tabacum* leaf disk method. The overexpressed calli was induced by infection of *P. mume* ‘Mu Guamei’ leaves, and qRT-PCR was performed under sterile conditions to screen transgenic positive lines.

### Transient gene expression in *P. mume* leaves

Transiently overexpressing *PmNAC32* was performed using the 1301-PmNAC32 constructs. For Virus Induced Gene Silencing (VIGS), a gene-specific fragment of *PmNAC32* (707–898 bp) was inserted into the pTRV2 vector to produce the pTRV2-*PmNAC32* constructs. Transient transformation experiments were executed as previously specified with some modifications [82]. The constructs were then transformed into *A. tumefaciens* strain GV3101. Single bacteria were isolated into 50 ml of Luria–Bertani broth containing 50 mg/l Kanamycin and Rifampicin and cultured at 220 rpm in an incubator at 28°C until optical density at 600 nm of 0.8 was achieved. After centrifugation, the cell pellet was resuspended in 50 ml of hormone-free, uncontrolled pH MS liquid medium for 4 h. The 4-month-old seedlings of the *P. mume* variety ‘Longyan’ were inverted and submerged in the resuspension solution, followed by vacuum infiltration for 10 min. After blotting dry to remove residual liquid on the surface, the seedlings were cultured under dark conditions for 1 day before being transferred to a light incubator (16 h light/8 h dark). Leaves were collected after 3 days of cultivation for qRT-PCR analysis.

### RNA isolation, cDNA synthesis, and qRT-PCR

Total RNA was isolated from 0.4 g leaf tissue using an RNA-prepared Pure Plant Kit (Foregene, Chengdu, China). RNA integrity was assessed by Agilent 2100 Bioanalyzer (Agilent Technologies, Palo Alto, USA), and RNA quality was evaluated by gel electrophoresis. The ABI Quant Studio 5 real-time PCR system (Applied Biosystems, Foster City, USA) and the SYBR Green Real-Time PCR Master Mix (Toyobo, Osaka, Japan) were used for qRT-PCR. Firststrand cDNA was generated using the PrimeScript RT Reagent Kit with gDNA Eraser (Takara Bio, Shiga, Japan). Each analysis included three biological replicates. The *tubulin β-8 chain-like* (*TUB8*) gene of *P. mume* was employed as an internal reference in *P. mume*, the *tubulin β-8 chain-like* (*TUB8*) gene of *N. tabacum* was used as an internal control in *N. tabacum*, and the *actin2* gene of *A. thaliana* was used as an internal control in *A. thaliana*. The 2^−ΔΔCT^ method was used to calculate relative expression level. Data analysis was performed with GraphPad Prism 9.0.0. The qRT-PCR primer sequences are provided in the appendix (Table S2).

### Protein extraction and western blot

The antibody against the PmNAC32 protein was used for immunoblotting at 1:1000 dilution (ABclonal Technology, Wuhan, China). PmNAC32 protein-specific polypeptide (160–283 amino acids [aa]) was selected as the immunogen, and ABclonal Technology produced the anti-PmNAC32 polyclonal antibody. To detect the expression of PmNAC32 in *P. mume* along with the diurnal rhythm, both the LD- and SD-treated *P. mume* seedlings were sampled from 0 to 24 h every 4 h and then western blot was performed (Protein marker 20351ES#, Yeasen Biotechnology, Shanghai, China) using Actin (26F7) mAb as control (M20009#, Abmart, Shanghai, China).

### Yeast two-hybrid assay

Three ORFs and six truncated fragments (PmNAC32-N: 1-191aa; PmNAC32-C: 192-320aa; PmNAC47-N: 1-184aa; PmNAC47-C: 185-315aa; PmNAC29-N: 1-179aa; PmNAC29-C: 180-302aa) were inserted into the pGBKT7 (BD) vectors (Table S1). The recombinant plasmids were transformed into *Saccharomyces cerevisiae* strain Y2HGold (Weidi Biotechnology, Xining, China), according to the manufacturer’s guidelines. Yeast cells were cultured at 28°C for 3 days on DDO (SD medium without leucine and tryptophan) media and then transferred to QDO (SD medium without leucine, tryptophan, histidine, and adenine) plates containing X-α-Gal. The ORFs of *PmNAC32*, *PmNAC47*, and *PmNAC29* were inserted into the pGADT7 (AD) vector. The AD-recombinants and BD-recombinants were cotransferred into Y2HGold yeast strains. The potential pair-to-pair interaction among PmNAC32, PmNAC47, and PmNAC29 was verified on QDO + X-α-Gal.

### Pull-down assay

The *PmNAC32* and *PmNAC47* ORFs were inserted into the pCold-Trigger Factor (pCold-TF) vector to generate His-PmNAC32 and His-PmNAC47 construct, and the ORFs of *PmNAC29* and *PmNAC47* were inserted into the pMAL-c2x vector to create MBP-PmNAC29 and MBP-PmNAC47 recombinants, utilizing specific primers for amplification (Table S1). His-tagged and GST-tagged recombinant proteins were produced in *Escherichia coli* Rosetta (DE3) by induction with 0.5 mM isopropyl *β*-D-thiogalactoside. MBP-PmNAC29 and MBP-PmNAC47 were incubated with immobilized His-empty, His-PmNAC32, or His-PmNAC47. Anti-MBP antibody and anti-His antibody (Sangon Biotech, Shanghai, China) were employed for immunoblotting analysis to verify protein interactions.

### Bimolecular fluorescence complementation assay

To perform BiFC assays, the ORFs of *PmNAC32* and *PmNAC29* were cloned into plant expression vectors containing eYFPN173 fusions to generate pSPYNE-PmNAC32 and pSPYNE-PmNAC29 (Table S1). And the ORFs of *PmNAC29* and *PmNAC47* were cloned into plant expression vectors containing eYFPC156 fusions to generate pSPYCE-PmNAC29 and pSPYCE-PmNAC47. The recombinant plasmids were transferred into *A. tumefaciens* strain GV3101. The inner epidermal cells of the onion were soaked in the *Agrobacterium* suspensions containing OD600 of 0.6 for 2–3 min, and the soaked inner epidermis of onion was placed on MS medium and cultured under dark conditions at 28°C for 2–3 days. BiFC fluorescence localization was visualized using a confocal laser scanning microscope (LSM800, Zeiss, Oberkochen, Germany).

### Yeast one-hybrid assay

The Y1H assay employed the AD-PmNAC32 constructs from the yeast two-hybrid (Y2H) assay. The promoter segment with NAC binding site (NACBS) of *PmCOL5* (−1433 to −1147 bp) was denoted as P1; a *PmCOL4* promoter fragment (−1003 to −798 bp) was denoted as P2. P1 and P2 were constructed on pAbAi vector to obtain the constructs (Table S1). Subsequently, P1/P2 were cultured on an SD/-Ura medium after being converted into the Y1H yeast strain. After the positive colonies were confirmed, AD-Empty and AD-PmNAC32 were transferred into positive bacteria and coated on an SD/−Leu + AbA (SD medium without leucine with Aureobasidin A) medium. The ORFs of *PmLHY*, *PmLHY-like*, *PmRVE1*, *PmRVE3*, *PmRVE6*, *PmRVE7*, and *PmRVE8* were cloned into AD vector. The promoter fragment containing EE-box of *PmNAC32* (−1476 to −1688 bp) was denoted as *PmNAC32*pro (EE); the promoter fragment containing CBS-box of *PmNAC32* (−214 to −324 bp) was denoted as *PmNAC32*pro (CBS); both were constructed on pAbAi vector to obtain the constructs. Follow-up steps are described in the preceding section. The growth status of the strains determined the interaction between them.

### Electrophoretic mobility shift assay

Sangon Biotech synthesized the 5′ biotin labeling sequences containing the assumed binding sites in promoters with NACBS (4E1, 4E2, 4E3, 4E4, 5E1, 5E2) based on primer pairs in Fig. 6 and Fig. S10. They also synthesized the necessary FAM 5′ end labeling sequences containing the assumed binding sites in promoters *PmNAC32*pro (EE) and *PmNAC32*pro (CBS) based on the primer pairs in Fig. 2. Mutant probes and competitors were unlabeled probes. The protein uses the previously described His-PmNAC32, His-PmNAC29, and His-PmNAC47 proteins. In the single-protein assay, His-PmNAC32 protein was used at 2 μg, while in the protein complex assay, His-PmNAC32 was used at 1 μg, with His-PmNAC29 and His-PmNAC47 proteins each added at 0.5 μg. EMSA assays were conducted with the Chemiluminescent EMSA Kit (Beyotime Biotech, Shanghai, China). Resultant sample was loaded into a natural 6% polyacrylamide gel with 0.5 × TBE buffer. The film was electroprinted into nylon film (Merck Darmstadt, Germany). After UV cross-linking for 4 min, the membrane was submerged in blocking solution for 40 min and washed 4 times in 1 × washing buffer. Then, the chemiluminescence signal was visualized by a charge-coupled device (CCD) camera.

### Dual-luciferase reporter gene assay

The whole promoter length of *PmNAC32*, *PmCOL5*, and *PmCOL4* sequences design primers (Table S1) were amplified and constructed on pGreenII_0800-5_LUC vector to obtain *PmNAC32*pro::LUC, *PmCOL5*pro::LUC, and *PmCOL4*pro::LUC constructs, which were then transformed into *Agrobacterium* strain GV3101 (pSoup). As previously described, the suspended *Agrobacterium* was injected into the *N. tabacum* at a ratio of PmRVE1/3–1301: *PmNAC32*pro::LUC = 9:1, PmNAC32–1301: *PmCOL5*pro::LUC/ *PmCOL4*pro::LUC = 9:1, and PmNAC32–1301: PmNAC29–1301: PmNAC47–1301: *PmCOL5*pro::LUC/ *PmCOL4*pro::LUC = 3:3:3:1. According to published methods, fluorescence activity detection used a CCD camera and IndiGO software [83].

## Supplementary Material

Web_Material_uhaf157

## Data Availability

The data presented in this study are included in the manuscript or supplementary materials. The processed RNA-seq datasets have been deposited in the NCBI Sequence Read Archive (SRA; PRJNA1060359).
