# Supplementary material for: A NAC family gene PmNAC32 associated with photoperiod promotes flower induction in Prunus mume
Source: Hortic Res. 2025 Jun 23;12(9):uhaf157. doi: 10.1093/hr/uhaf157 (PMC12317187; doi:10.1093/hr/uhaf157)
Supplement: Web_Material_uhaf157 [file web_material_uhaf157.zip › Supplementary Figures-Revision.doc]

**Figure S1**

GO Enrichment Analysis of LDC1 and SDC12 clusters in *P. mume*. (A) Top 10 enriched GO terms among LDC1 cluster. (B) Top 10 enriched GO terms among SDC12 cluster.

**Figure S2**

(A) Transcription profiling of central oscillator genes. The transcriptional response to varying the photoperiod form the RNA-seq. The abscissa indicates the sampling time point. Values shown are means (n=2), and the error bars represent the SE. FPKM, fragments per kilobase of sequence per million mapped reads. (B) and (C) The 45 TFs Identified both in LDC1 and SDC12 clusters in 22 TF Families.

**Figure S3**

GUS staining of *PmNAC32*pro::GUS lines. (A-E) GUS staining of different tissues in *PmNAC32*pro::GUS lines. A: flower, B: leaves, C: pods, D: stems, E: roots. Bars in (A-E) = 1000 μm. (F) GUS staining of *PmNAC32*pro::GUS lines at different time points under LD condition. Bars in (F) = 1 cm.

**Figure S4**

Detection of *A. thaliana* and *N. tabacum* *PmNAC32*-OE lines and the phenotype of WT and *PmNAC32*-OE1-3 *A. thaliana* lines under SD conditions. (A) Detection of *PmNAC32* gene of WT and *PmNAC32*-OE1-3 lines. M, DNA Marker. (B) GUS staining of leaves from WT and *PmNAC32*-OE1-3 lines. (C) The transcript levels of *PmNAC32* in *PmNAC32*-OE lines were assessed by qRT-PCR. n/a means not applicable. Values are indicated as mean ± SE (n = 3). (D) Flowering time phenotype of 46-day-old WT and *PmNAC32*-OE1-3 lines. Bars in (D) = 5 cm. (E) Average bolting time of WT and *PmNAC32*-OE1-3 lines shown in (D). Values are indicated as mean ± SE (n = 15). (F) Average number of leaves of WT and *PmNAC32*-OE1-3 lines. Values are indicated as mean ± SE (n = 15). (G) Detection of *PmNAC32* gene of *N. tabacum* WT and *PmNAC32*-OE1-3 lines. M, DNA Marker. (H) GUS staining of leaves from *N. tabacum* WT and *PmNAC32*-OE1-3 lines. Student’s test identified significant differences from the control (*****P* < 0.0001).

**Figure S5**

(A) Average bolting time of pER8-*PmNAC32*+DMSO lines and pER8-*PmNAC32*+EST lines (B) qRT-PCR assay for quantifying the abundance of *PmNAC32* transcript after treatment. Bars in (A) and (B) = 5 cm. (C) Bolting time phenotype of 26-day-old plants of pER8-*PmNAC32*+DMSO lines and pER8-*PmNAC32*+EST lines. (D) The transcript levels of *PmNAC32* in pER8-*PmNAC32*+DMSO lines and pER8-*PmNAC32*+EST lines were assessed by qRT-PCR. Values are indicated as mean ± SE (n = 3). Stars above the bars indicate significant differences as determined by Student's test (**P*<0.05; ***P*<0.01; *****P*<0.0001).

**Figure S6**

qRT-PCR of *PmNAC32*, *PmCOL5*, *PmCOL4*, *PmLHY*, *PmRVE1* and *PmRVE3* in leaves after *PmNAC32* overexpression and VIGS treatment. The expression level of (A) *PmNAC32* (B) *PmCOL5* (C) *PmCOL4* (D) *PmLHY* (E) *PmRVE1* (F) *PmRVE3* after *PmNAC32* overexpression. The expression level of (A) *PmNAC32* (B) *PmCOL5* (C) *PmCOL4* (D) *PmLHY* (E) *PmRVE1* (F) *PmRVE3* after *PmNAC32* VIGS treatment. Values are indicated as mean ± SE (n = 3). Stars above the bars indicate significant differences as determined by Student's test (ns>0.05; ***P*<0.01; ****P*<0.001, *****P*<0.0001).

**Figure S7**

Analysis of photoresponsive cis-elements in *PmNAC32* promoters. Red stands for circadian; yellow for G-box; green for CBS-box; blue for EE-box.

**Figure S8**

(A) and (B) Interactions of PmRVEs with the *PmNAC32*pro(EE) and *PmNAC32*pro(CBS) were detected in Y1H respectively. AD represents the negative control.

**Figure S9**

The expression levels of *PmRVE1*, *PmRVE3* and *PmLHY* in *PmNAC32*-OE calli were detected by qRT-PCR. (A) *PmRVE1* (B) *PmRVE3* (C) *PmLHY*. Values are indicated as mean ± SE (n = 3). Stars above the bars indicate significant differences as determined by Student's test (*ns*>0.05; **P*<0.05; ***P*<0.01; ****P*<0.001; *****P*<0.0001;).

**Figure S10**

His-PmNAC32 recombinant protein can bind NACBS in *PmCOL4* and *PmCOL5* promoter. (A)4E2; (B)4E3; (C) 4E4; (D) 5E2. DNA probes labeled with 5’ Biotin. Same but unlabeled DNA probes were used as competitors. Different but unlabeled DNA probes were used as mutators.

**Figure S11**

Detection of *A. thaliana* *PmCOL4*-OE and *PmCOL5*-OE lines (A) Detection of *PmCOL4* gene in WT and *PmCOL4*-OE1-3 lines. M, DNA Marker. (B) GUS staining of leaves from WT and *PmCOL4*-OE1-3 lines. (C) The transcript levels of *PmCOL4* in *PmCOL4*-OE lines were assessed by qRT-PCR. Values are indicated as mean ± SE (n = 3). (D) Detection of *PmCOL5* gene in WT and *PmCOL5*-OE1-3 lines. M, DNA Marker. (E) GUS staining of leaves from WT and *PmCOL5*-OE1-3 lines. (F) The transcript levels of *PmCOL5* in *PmCOL5*-OE lines were assessed by qRT-PCR. Values are indicated as mean ± SE (n = 3). n/a means not applicable.

**Figure S12**

Alignment of the cloned amino acid sequences of PmNAC32, PmNAC29 and PmNAC47 proteins. The amino acid sequences of PmNAC32, PmNAC29 and PmNAC47 were aligned by Clustal X program. The amino acid residues conserved among the proteins are highlighted in black. The different amino acid residues are indicated with an asterisk*. The red frame indicated the conserved NAM domain.

**Figure S13**

Detection of *A. thaliana* *PmNAC29*-OE and *PmNAC47*-OE lines and the phenotype of *PmNAC29*-OE1-3 and *PmNAC47*-OE1-3 under SD conditions. (A) Detection of *PmNAC29* in WT and *PmNAC29*-OE1-3 lines. M, DNA Marker. (B) GUS staining of leaves from WT and *PmNAC29*-OE1-3 lines. (C) The transcript levels of *PmNAC29* in *PmNAC29*-OE lines were assessed by qRT-PCR. Values are indicated as mean ± SE (n = 3). (D) Detection of *PmNAC47* in WT and *PmNAC47*-OE1-3 lines. M, DNA Marker. (E) GUS staining of leaves from WT and *PmNAC47*-OE1-3 lines. (F) The transcript levels of *PmNAC47* in *PmNAC47*-OE lines were assessed by qRT-PCR. Values are indicated as mean ± SE (n = 3). (G) Flowering time phenotype of 46-day-old WT and *PmNAC29*-OE1-3 lines. Bars in (G) = 5 cm. (H) Average bolting time of WT and *PmNAC29*-OE1-3 lines shown in (G). Values are indicated as mean ± SE (n = 15). (I) Average number of leaves of WT and *PmNAC29*-OE1-3 lines. Values are indicated as mean ± SE (n = 15). (J) Flowering time phenotype of 46-day-old WT and *PmNAC47*-OE1-3 lines. Bars in (J) = 5 cm. (K) Average bolting time of WT and *PmNAC47*-OE1-3 lines shown in (J). Values are indicated as mean ± SE (n = 15). (L) Average number of leaves of WT and *PmNAC47*-OE1-3 lines. Values are indicated as mean ± SE (n = 15). Stars above the bars show significant differences by Student’s test (*****P* < 0.0001).

**Figure S14**

Transcriptional activation detection. (A) *PmNAC32* (B) *PmNAC29* (C) *PmNAC47*. The left part depicts the tested regions of the gene.
